# The NF-κB Activating Pathways in Multiple Myeloma

**DOI:** 10.3390/biomedicines6020059

**Published:** 2018-05-16

**Authors:** Payel Roy, Uday Aditya Sarkar, Soumen Basak

**Affiliations:** Systems Immunology Laboratory, National Institute of Immunology, Aruna Asaf Ali Marg, New Delhi 110067, India; payelroy87@nii.ac.in (P.R.); udayaditya2992@nii.ac.in (U.A.S.)

**Keywords:** NF-κB, multiple myeloma, canonical, non-canonical, mutations, microenvironment, cytokines, crosstalk, gene-expressions

## Abstract

Multiple myeloma(MM), an incurable plasma cell cancer, represents the second most prevalent hematological malignancy. Deregulated activity of the nuclear factor kappaB (NF-κB) family of transcription factors has been implicated in the pathogenesis of multiple myeloma. Tumor microenvironment-derived cytokines and cancer-associated genetic mutations signal through the canonical as well as the non-canonical arms to activate the NF-κB system in myeloma cells. In fact, frequent engagement of both the NF-κB pathways constitutes a distinguishing characteristic of myeloma. In turn, NF-κB signaling promotes proliferation, survival and drug-resistance of myeloma cells. In this review article, we catalog NF-κB activating genetic mutations and microenvironmental cues associated with multiple myeloma. We then describe how the individual canonical and non-canonical pathways transduce signals and contribute towards NF-κB -driven gene-expressions in healthy and malignant cells. Furthermore, we discuss signaling crosstalk between concomitantly triggered NF-κB pathways, and its plausible implication for anomalous NF-κB activation and NF-κB driven pro-survival gene-expressions in multiple myeloma. Finally, we propose that mechanistic understanding of NF-κB deregulations may provide for improved therapeutic and prognostic tools in multiple myeloma.

## 1. General Introduction

Heterogeneous cancer-associated mutations often influence the interaction of malignant cells with their microenvironment that modifies therapeutic outcomes. Therefore, an understanding of the molecular mechanism underlying the coordinated functioning of genetic mutations and microenvironmental cues may have significance for the effective management of neoplastic diseases. Within the tumor microenvironment, immune cells as well as stromal cells secrete a diverse array of pro-inflammatory cytokines, which activate key pro-survival signaling pathways in malignant cells. Of particular importance is the NF-κB system, which forms a major link between cancer and inflammation. Interestingly, sequencing of cancer genomes revealed recurrent gain-of-function mutations in genes encoding key positive regulators of NF-κB signaling and inactivating genetic aberrations in negative regulators of this pathway. Multiple myeloma (MM), a plasma cell malignancy, provides one of the best examples where a number of mutations have been mapped onto the NF-κB pathway. In addition, microenvironment-derived signals were also shown to modulate NF-κB-dependent gene expressions in myeloma cells. Here, we briefly review multiple myeloma and the NF-κB signaling system. We then discuss the NF-κB-activating genetic lesions associated with MM and the role of the tumor microenvironment in reinforcing NF-κB signaling in cancerous cells. Finally, we elaborate interdependent regulations of NF-κB-activating pathways in MM. 

## 2. Multiple Myeloma—Epidemiology and Aetiology

Multiple myeloma is the second most widespread hematologic malignancy after non-Hodgkin lymphoma, with a global estimate of 103,826 new cases and 72,453 mortalities annually [1]. The disease is more prevalent in men than in women, and the median age at diagnosis is 66 years [2]. The disease incidence also varies with ethnicity, being more prevalent among Caucasians than in Asians [1]. The American Cancer Society estimates that about 30,770 individuals (16,400 men and 14,370 women) will be diagnosed with this disease in the USA in 2018 (https://cancerstatisticscenter.cancer.org/#!/cancer-site/Myeloma; accessed on 10 March 2018). Indeed, it has been found that the incidence and the mortality rate are significantly higher in African Americans as compared to their Caucasian counterparts [3]. Various studies estimate that the incidence of MM in India is around 1.2–1.8 per 100,000 individuals. 

**Figure 1:**
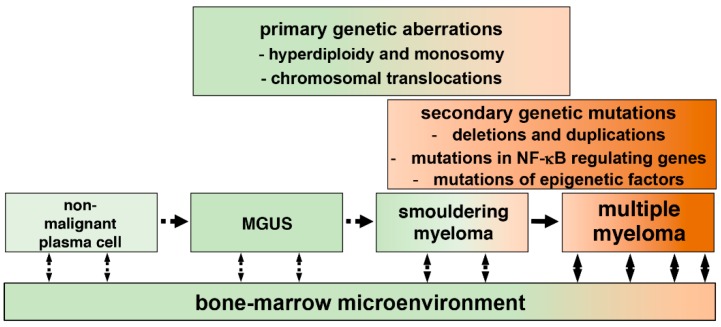
Development of multiple myeloma (MM) from plasma cells. Progression of MM from post-germinal center plasma cells to symptomatic myeloma occurs through intermediate MGUS (monoclonal gammopathy of undetermined significance), and SMM (smouldering MM) stages. Plasma cells associated with MGUS and SMM may display chromosomal abnormalities, which trigger cellular transformation. Cancerous plasma cells acquire additional, secondary genetic mutations, which support tumor growth by activating key signaling pathways in malignant cells. Finally, cell–cell communications, which involve physical interactions between cancerous cells and cancer-associated stromal cells, and secretion of cytokines and other soluble factors, promote survival and proliferation of myeloma cells within the bone marrow microenvironment.

MM is characterized by the clonal proliferation of cancerous plasma cells (PCs) in the bone marrow microenvironment and an associated increase in the level of monoclonal (M) protein in blood and serum [4]. Myeloma cells are characterized by high rates of somatic hypermutation of immunoglobulin (Ig) genes and isotype class switching, but differ from healthy PCs with respect to the abundance of certain cell surface molecules, including CD138 and CD38 [5,6]. In most cases, MM develops from monoclonal gammopathy of undetermined significance (MGUS) and smouldering MM (SMM) (Figure 1), conditions that involve high levels of M-protein and bone marrow (BM) plasmacytosis [7]. Clinical manifestations of MM include lytic bone lesions, hypercalcemia, cytopenia, renal dysfunction, hyperviscosity and peripheral neuropathy [8].

Therapeutic intervention for MM currently involves six categories of medication—(1) immunomodulatory drugs (IMiDs) such as lenalidomide and pomalidomide; (2) proteasome inhibitors (PIs) such as bortezomib, carfilzomib, and ixazomib; (3) histone deacetylase inhibitors such as panobinostat; (4) monoclonal antibodies (MAbs) such as daratumumab and elotuzumab; (5) DNA alkylating agents; and (6) glucocorticosteroids [9]. Recent advancement in the management of the disease led to a substantial improvement in the 5-year survival rate in MM. The 5-year relative survival percent reported in the SEER database of the National Cancer Institute, USA steadily improved from 24.6% in between 1975–1977 to 52.4% in between 2008–2014 (https://seer.cancer.gov/csr/1975_2015/browse_csr.php?sectionSEL=18&pageSEL=sect_18_table.08.html; accessed on 14 May 2018). The disease, however, remains incurable because of widespread drug-resistance and relapse in most patients.

Although it is difficult to pinpoint the exact trigger of MM, karyotyping and DNA sequencing studies involving patient-derived cells or MMCLs identified several genomic translocations and mutations (Table 1 and Figure 1). The frequency and extent of these genetic aberrations were substantially augmented in individuals with advanced-stage disease or poor prognosis and in those with refractory MM unresponsive to therapy [10,11,12,13]. Nearly half of the MM tumors are hyperdiploid, characterized by trisomies of chromosomes 3, 5, 7, 9, 11, 15, 19 and 21 [14]. The non-hyperdiploid MM is associated with chromosomal translocations involving the immunoglobulin heavy-chain (IgH) locus and the loci encoding MMSET, FGFR3, CCND3 (cyclin D3), CCND1 (cyclin D1), MAF (c-Maf) or MAFB [15,16,17,18,19,20]. Moreover, duplications involving chromosome 1 and deletions affecting several other chromosomal arms have been associated with the onset of the disease [21,22,23]. Mutational events secondary to oncogenic transformation have also been reported in MM [24]. For example, frequent mutations have been observed in RAS-encoding *NRAS* and *KRAS* genes that triggered aberrant MAPK activity [25]. In addition, secondary mutations were mapped onto genes implicated in NF-κB regulations (discussed later), *CDKN2C* that encodes cyclin-dependent kinase inhibitor 2C [26], *TP53* expressing the tumor suppressor p53 [27] and *MYC* that codes for the proto-oncogene cMyc [28]. Disease progression is also influenced by physical or cytokine-mediated interactions between myeloma cells and the bone-marrow stromal cells. These cell-to-cell communications further inform the proliferative and anti-apoptotic program by modulating the NF-κB activity in myeloma cells.

The complex mutational landscape, which produces bioclinical heterogeneity, presents a significant challenge in the management of multiple myeloma. Risk-prediction based on traditional biomarkers measured using solely protein analysis and conventional cytologic assays often do not match with the actual patient outcomes and exhibit poor correlation with the minimal residual disease (MRD). Cutting-edge next-generation sequencing (NGS) technology has now made it possible to acquire a more comprehensive view of genomic alterations in MM. NGS not only offers genome-scale data but can also detect very low-frequency mutations [29]. Not surprisingly, NGS-based methods substantially improved the sensitivity of MRD detection in MM [30]. Furthermore, NGS provides for reliable measurement involving both bone-marrow samples and blood biopsies, enabling non-invasive diagnosis of MM. In fact, these high-throughput sequencing technologies helped to define the mutational landscape of bone-marrow resident as well as circulating myeloma cells at single-cell resolution [31]. As discussed later, NGS-based studies were also instrumental in charting NF-κB deregulating mutations in MM. In this article, we have focused on NF-κB deregulations in MM; for a more comprehensive description of MM and the underlying genetic as well as cell-signaling anomalies, please see [4,32].

## 3. The NF-κB Signaling System

The nuclear factor kappa B (NF-κB) system functions in a wide variety of cells and coordinates innate and adaptive immune responses. Not surprisingly therefore, deregulated NF-κB activities have been implicated in several human ailments, including hematologic cancers [36,37]. The NF-κB family consists of five structurally related monomeric subunits: RelA (also called p65), RelB, c-Rel, p50 (encoded by *NFKB1* and produced as a precursor protein p105) and p52 (encoded by *NFKB2* and produced as a precursor p100). Combinatorial association of the mature subunits generate 15 possible homo- or heterodimeric transcription factors, with the most prevalent being the RelA:p50 and the RelB:p52 dimers. The NF-κB proteins possess a conserved Rel homology region (RHR) in their N-termini that contains the domains for dimerization and DNA binding as well as a nuclear localization sequence (NLS). NF-κB dimers recognize a broad consensus DNA sequence known as the κB motif, which is represented as: 5′-GGRN(W)YYCC-3′ (where R denotes A or G; N denotes A, C, G or T; W denotes an A or T; Y denotes T or C) [38]. In resting cells, NF-κB factors are held inactive in the cytoplasm by inhibitor proteins. Extracellular stimuli trigger the canonical (also known as classical) or the non-canonical (also known as alternative) NF-κB pathway to induce translocation of NF-κB dimers into the nucleus, where they mediate the expression of hundreds of immune and stress response genes as well as immune-differentiating and pro-survival factors.

## 4. The Canonical NF-κB Activation Pathway

**Figure 2:**
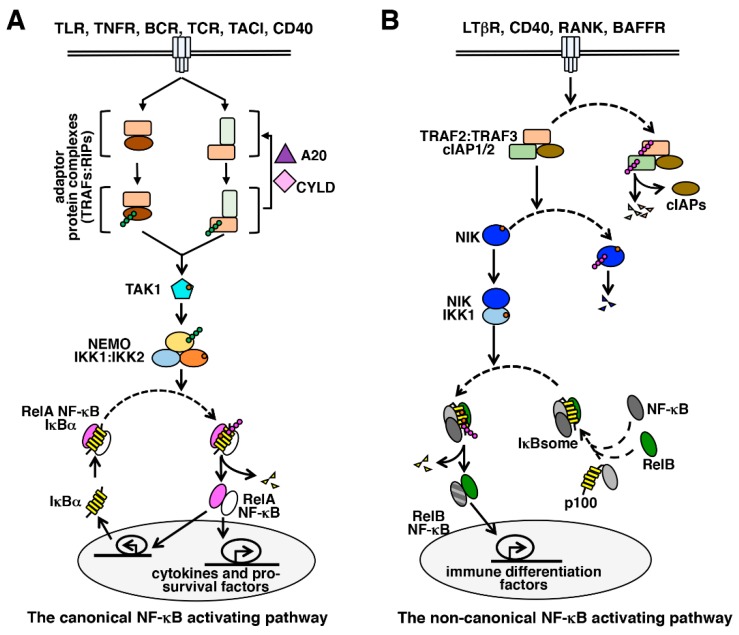
NF-κB activating pathways. The NF-κB system consists of (**A**) the canonical and (**B**) the non-canonical pathways. In general, immune-activating cues activate the canonical pathway, which stimulates the synthesis of pro-inflammatory and pro-survival factors involving the RelA NF-κB activity; and non-canonical signaling triggers RelB NF-κB activation during immune differentiation.

Activation of canonical NF-κB signaling: inhibitory IκB proteins, including the major isoform IκBα as well as IκBβ and IκBε, sequester pre-existing NF-κB dimers in the cytoplasm of unstimulated cells [39]. Signals emanating from cytokine receptors such as tumor necrosis factor receptor-1 (TNFR1), pathogen-sensing receptors such as Toll-like receptors (TLRs), and B- or T-cell antigen receptor (BCR or TCR) engage the canonical NF-κB pathway to activate NF-κB dimers from the IκB-inhibited complexes (Figure 2A). Central to this pathway is the trimeric IκB kinase (IKK) complex, which is comprised of two catalytic subunits IKK1 (also known as IKKα) and IKK2 (also known as IKKβ) and a regulatory subunit NEMO (also known as IKKγ). However, the enzymatic activity of IKK2, and not IKK1, was found to be essential for triggering of the canonical pathway. Intracellular adaptor proteins belonging to the receptor-interacting serine/threonine-protein (RIP) and TNF receptor-associated factor (TRAF) families promote signal-induced activation of TGFβ activated kinase-1 (TAK1), which in turn phosphorylates and activates IKK2. In particular, K63-linked polyubiquitination of TRAF2 and TRAF6 as well as NEMO nucleates the assembly of a receptor-proximal complex, which coordinates the activation of upstream kinases of the canonical NF-κB pathway [40]. Indeed, deubiquitinating enzymes such as CYLD and A20 function as important negative regulators of NF-κB signaling. The activated IKK complex, in turn, phosphorylates IκBα, IκBβ and IκBε that induce K48-linked polyubiquitination and proteasomal degradation of these classical IκBs, and consequent release of the bound NF-κB dimers into the nucleus. 

**Target genes of the canonical NF-κB pathway:** in most cell-types, canonical signaling activates RelA:p50 and c-Rel:p50 heterodimers. Once in the nucleus, these NF-κB heterodimers induce the transcription of an array of genes (see http://www.bu.edu/nf-kb/ for a complete list). Importantly, IκBα itself is encoded by an NF-κB target gene; NF-κB-dependent re-synthesis of IκBα constitutes a negative feedback loop that entails sequestration and nuclear export of NF-κB dimers by IκBα and post-induction attenuation of the canonical NF-κB response. Indeed, coordinated degradation and re-synthesis of classical IκBs allow a tight control over the amplitude and the duration of the NF-κB activity induced in the canonical pathway [41]. There are several other review articles, including that of Mitchell et al. [41], that provide further details of the regulatory mechanisms underlying the activation and post-induction attenuation of canonical NF-κB signaling.

Other NF-κB-target genes encode tumor-promoting, pro-inflammatory cytokines such as TNF, IL-1 and IL-6 (Interleukin 6); growth-stimulating cytokines and factors such as IL-2, GM-CSF (granulocyte-macrophage colony-stimulating factor), M-CSF and CD40L; cell-adhesion molecules such as ICAM-1 (intercellular adhesion molecule-1) and stress response mediators such as iNOS (inducible nitric oxide synthase). In addition, NF-κB factors also modulate cell-cycle progression by mediating the expression of cell-cycle regulators, such as cyclin D1. Notably, NF-κB proteins promote cell survival by triggering the expression of genes encoding anti-apoptotic proteins, such as cFLIP (cellular FLICE (FADD-like IL-1β-converting enzyme)-inhibitory protein), Bcl-2, Bcl-xL, XIAP, cIAPs (cellular inhibitors of apoptosis) and survivin. Indeed, RelA was shown to play a critical role in protecting cells from apoptotic death [39]. The canonical NF-κB signaling has been implicated in both innate and adaptive immune responses to a variety of microbial substances. In particular, B cell maturation antigen (BCMA), which activates the canonical NF-κB pathway, was shown to be required for the maintenance of long-lived plasma cells [42,43]. Previous studies, in fact, identified NF-κB transcriptional signatures in plasma cells isolated from the bone marrow of healthy individuals [34]. Moreover, canonical signaling supported the survival of plasma cells *in vitro* [44]. These observations indicated possible engagement of the canonical pathway in normal bone-resident plasma cells in physiological settings. 

## 5. The Non-Canonical NF-κB Activation Pathway

Activation of non-canonical NF-κB signaling: the non-canonical NF-κB pathway is activated by cell-differentiating and organogenic cues, that engage B-cell maturating BAFFR (B cell activating factor receptor), CD40 (cluster of differentiation 40), osteoclast differentiating RANK (receptor activator of NF-κB) or lymph node-inducing lymphotoxin-β receptor (LTβR) [45]. The *Nfkb2*-encoded NF-κB molecule p100 regulates nuclear activation of RelB NF-κB dimers in the non-canonical pathway (Figure 2B). As a constituent of the multimeric IκBsome complex, p100 utilizes its C-terminal ankyrin repeat domain for sequestering primarily the RelB NF-κB factors in the cytoplasm of unstimulated cells [46,47]. Non-canonical signaling requires phosphorylation and activation of IKK1 by NF-κB inducing kinase (NIK) and does not involve IKK2 or NEMO [48]. NIK, in association with activated IKK1, phosphorylates p100 [48,49,50]. Subsequent K48-linked polyubiquitination and proteasomal action removes the C-terminal inhibitory domain of p100 that not only generates the mature p52 NF-κB subunit but also liberates the RelB:p52 and the RelB:p50 NF-κB heterodimers from the IκBsome into the nucleus [51]. Unlike canonical signaling, which triggers a strong yet transient RelA or c-Rel containing NF-κB response, non-canonical signaling elicits a sustained RelB NF-κB activity. In physiological settings, RelB activated by non-canonical signaling isthought to play a rather limited role in immune cell maturation and immune organogenesis, including B-cell maturation, dendritic cell activation, bone homeostasis and lymphoid organogenesis. Importantly, IKK1 and RelB as well as p100 have been implicated in the survival of post-germinal center plasma cells [52,53]. If the non-canonical NF-κB pathway modulates the survival of long-lived bone marrow plasma cells, the healthy counterpart of myeloma cells, remains unclear.

Interestingly, NIK is regulated at the level of protein stability. A complex composed of TRAF3, TRAF2 and cIAP1/2 targets NIK for K48-linked polyubiquitination that causes proteasomal degradation of NIK in resting cells. Non-canonical stimuli degrade TRAF3 and TRAF2 and rescue NIK from the constitutive destruction; NIK then marks p100 for proteasome processing [54,55]. In addition, it was proposed that IKK1-mediated phosphorylation triggers proteasomal degradation of NIK and consequent termination of non-canonical signaling [56].

**Target genes of RelB NF-κB:** while immune-activating substances trigger canonical signaling, immune-differentiating cues signal through the non-canonical pathway. RelA or c-creRel dimers activated by canonical signaling induce the expression of a wide spectrum of immune and stress response genes as well as pro-survival factors. It was originally suggested that RelB NF-κB dimers activated by the non-canonical pathway induce preferentially the expression of genes encoding organogenic chemokines and immune differentiating factors [45]. Indeed, the RelB:p52 dimer was shown to bind to the variant kappaB site present in the promoters of homeostatic chemokine genes, including stromal cell-derived factor 1α (SDF1α), and selectively activate their expressions [57,58,59]. Accordingly, it was postulated that stimulus-selective gene-expressions are achieved through the engagement of distinct NF-κB dimers, which possess non-redundant DNA binding specificity [38]. However, this notion has been contested by several other studies. In fact, protein-binding microarrays (PBMs) and surface plasmon resonance (SPR) analyses indicated that RelA:p50, c-Rel:p50 and RelB:p52 heterodimers not only bind to identical κB sites, but they also show similar affinities towards DNA [60]. Moreover, ChIP-seq analyses demonstrated that various NF-κB subunits occupy equivalent chromatin sites *in vivo* [61,62]. Finally, RelB was shown to activate the expression of at least a subset of RelA-target genes, including those encoding pro-inflammatory cytokines and pro-survival factors, in mouse-derived knockout cells, specialized immune cells, and cancerous cells [63,64,65]. Therefore, regulatory mechanisms separate from DNA binding appear to dictate stimulus-specific expressions of NF-κB-dependent genes [66]. It is likely that dynamical control of the activated NF-κB dimers, signal-induced chromatin modifying events, other co-regulated transcription factors and their potentially different interaction with NF-κB proteins impact stimulus specific gene-expressions. Clearly, additional studies are warranted for unraveling how these individual NF-κB-activating pathways direct specific gene-expression programs in physiological settings. For further reading on non-canonical NF-κB signaling, please see other review articles including that of Shao-Cong Sun [45].

## 6. NF-κB Deregulating Mutations in Multiple Myeloma

As early as in 2007, two research groups independently reported the presence of NF-κB-activating mutations in MM [33,34]. Annunziata et al. observed NF-κB-related genetic anomalies in 28% of the MM cell lines (MMCL) and 9% of the primary MM tumors [33]. Keats et al. examined 155 MM samples using high-resolution array-based comparative genomic hybridization (aCGH) and gene expression profiling (GEP) [34]. Their study mapped close to 20% of the mutational events onto the genes encoding the mediators and effectors of NF-κB signaling. These pioneering studies identified MM-associated mutations in both canonical and non-canonical arms of the NF-κB system (Table 1). The most common genetic abnormalities affecting the canonical pathway were: gain-of-function mutations in the gene encoding the TACI receptor (transmembrane activator and CAML interactor) that mediates canonical signaling; homozygous deletions of *CYLD*; and amplifications of *NFKB1*, which produces p50. Frequently occurring genetic aberrations pertinent to the non-canonical pathway included gain-of-function mutations and amplifications of genes encoding LTβR, CD40 and NIK; frameshift mutations and deletions associated with *NFKB2* that resulted in the production of a truncated p100 devoid of the C-terminal inhibitory domain; and mutational inactivation or deletion of genes encoding negative regulators, such as TRAF2, TRAF3, cIAP1 and cIAP2.

Subsequent studies substantiated the prevalence of NF-κB-activating mutations in MM. Parallel sequencing of 38 tumor genomes catalogued 10 point-mutations and four structural rearrangements associated with 11 distinct genes, which directly or indirectly control NF-κB functions [67]. The deregulated genes linked to canonical signaling included *TLR4*; *TNFRSF1A*, which encodes TNFR1, *IKBKB*, which expresses IKK2; *IKBIP*, which encodes an IKK2-interacting protein; *CARD11, MAP3K1* and *RIPK4* that code for various IKK2-activating proteins; *CYLD*; *BTRC*, which encodes a ubiquitin ligase involved in signal-induced degradation of IκBα. The mutated genes encoding non-canonical signal transducers included *MAP3K14*, which produces NIK, and *TRAF3*. In a separate study, samples derived from 177 and 26 myeloma patients were subjected to whole-exome and whole-genome sequencing, respectively [25]. This high-throughput analysis identified homozygous deletions in 32 genes, including *CYLD* and *TRAF3*. Similarly, 17% of the 463 patients enrolled in the National Cancer Research Institute (UK) Myeloma XI trial possessed NF-κB-activating mutations, which affected most frequently *CYLD* and *TRAF3* [68]. Finally, a recent study identified recurrent loss-of-function mutations in the gene encoding the negative regulator of IKK2, A20 [35].

These mutations are associated with a diverse set of genes, but result invariably in the pathological activation of a handful of key signaling pathways, particularly the NF-κB-activating pathways. Therefore, it is tempting to speculate that an “NF-κB-high phenotype,” and not any particular genetic lesion, is enriched in MM and exacerbates the disease pathogenesis. MM-associated mutations potentially promote the nuclear activity of RelA and RelB heterodimers and trigger NF-κB-driven gene expressions. As such, heightened NF-κB activity has been implicated in the growth of myeloma cells, and in their resilience against apoptotic insults. Indeed, gene expression profiling of hyperdiploid multiple myeloma revealed a distinct patient cluster characterized by the overexpression of NF-κB-target genes, including anti-apoptotic genes [69]. Curiously, patients belonging to this NF-κB-signature cluster responded substantially better to bortezomib, a proteasome inhibitor that blocks degradation of IκBs, as compared to other patient groups (70% versus 29%; *p* = 0.02). This study underscored the importance of mutational activation of NF-κB signaling in the pathogenesis of MM. 

## 7. NF-κB-Related Microenvironmental Cues in Multiple Myeloma

Unlike most other hematological malignancies, development of symptomatic myeloma obligatorily involves a tumor-promoting microenvironment that is provided by the bone marrow niche [70,71]. Within the bone marrow microenvironment, myeloma cells interact with accessory cells, including bone marrow stromal cells (BMSCs), osteoclasts, osteoblasts and endothelial cells. These physical contacts provoke tumor-associated non-cancerous cells to produce various soluble factors, including cytokines and chemokines. Cell–cell communications involving physical interactions as well as soluble mediators, which engage autocrine and paracrine loops, activate NF-κB and other key signaling pathways in both non-malignant and malignant cells. Heightened NF-κB signaling leads to further accumulation of tumor-promoting cytokines and growth factors, which support growth, survival and drug-resistance of myeloma cells [70]. 

For example, IL-6 acts as an important growth and survival factor in MM. Upon adhering to myeloma cells, BMSCs activate the canonical NF-κB pathway, which induces the expression of IL-6 [72]. Additionally, myeloma cell-derived IL-1β, also encoded by an NF-κB-target gene, promotes IL-6 production by inducing canonical NF-κB signaling in BMSCs [73]. In turn, IL-6 binds to the cognate receptor and induces pro-proliferative and pro-survival gene-expressions in myeloma cells. Furthermore, IL-6 induces the production of vascular endothelial growth factor (VEGF), which promotes angiogenesis and neovascularization in the tumor microenvironment [74]. Importantly, some of the VEGF isoforms are encoded by NF-κB-target genes [75]. Bone marrow stromal cells also secrete insulin-like growth factor 1 (IGF1), which in myeloma cells induces NF-κB-dependent expression of anti-apoptotic genes [76,77]. Pro-inflammatory cytokine TNF activates canonical NF-κB signaling in both BMSCs as well as myeloma cells, and is also produced by the canonical pathway. TNF not only supports the pro-inflammatory tumor microenvironment but also induces the expression of important NF-κB-target pro-survival factors in myeloma cells [78]. Indeed, disruption of the TNF autocrine loop was shown to sensitize myeloma cells to apoptosis-inducing anticancer drugs ex vivo [79]. More so, it has been suggested that therapeutic efficacy of thalidomide and lenalidomide in MM in part relies on their ability to inhibit pro-inflammatory cytokines such as TNF-α, IL-1β, and IL-6 [80,81]. 

MM is also associated with an increased abundance of B-cell activating factor (BAFF), which activates non-canonical NF-κB signaling via BAFFR in addition to TACI-mediated induction of the canonical pathway [82]. As such, BAFF is thought to support growth and survival of myeloma cells and contribute to poor disease prognosis [83]. Importantly, a proliferation-inducing ligand (APRIL) also signals through TACI as well as BCMA, and constitutes an important bone-marrow microenvironmental factor in MM [70]. Of note, targeting of BCMA in chimeric antigen receptor T cell (CAR-T cell) based therapy of MM produced promising results in initial clinical trials [84]. Moreover, myeloma cells produce RANKL, which promotes MM-associated bone loss by inducing osteoclast differentiation through the non-canonical NF-κB pathway [85]. It was shown that physical interaction of myeloma cells with the extracellular matrix (ECM) component fibronectin *per se* induces a RelB-dependent, pro-survival NF-κB signaling in cancerous cells [86]. Finally, non-canonical signaling in BMSCs produce the RelB-target homeostatic chemokine SDF1α, which directs homing of myeloma cells to the bone marrow niche [87]. In sum, autocrinal as well as paracrinal cytokine signals, cell–cell and cell–ECM adhesion mechanisms seem to activate both canonical and non-canonical NF-κB signaling in myeloma cells as well as in tumor-associated non-malignant cells. 

## 8. Interactions between NF-κB Signaling Pathways and Multiple Myeloma 

**Figure 3:**
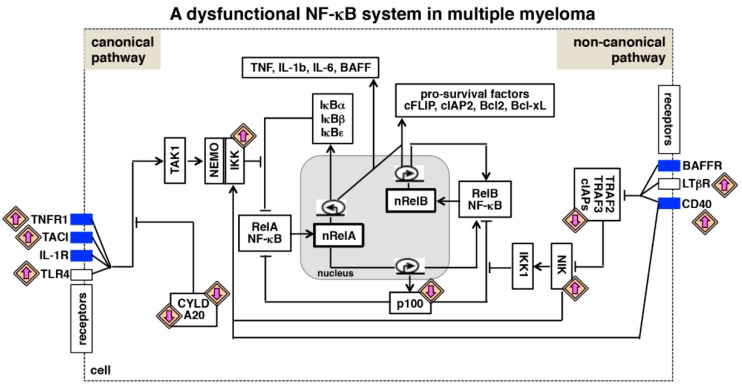
A dysfunctional NF-κB system in multiple myeloma. Various biochemical mechanisms interlink NF-κB-activating canonical and non-canonical pathways within an integrated NF-κB system. Also in myeloma cells, RelA and RelB containing dimers mediate expressions of overlapping set of anti-apoptotic genes. Multiple myeloma is associated with the gain-of-function mutation (upward directed purple arrow) of genes encoding NF-κB activators and the loss-of-function mutation (downward directed purple arrow) of genes encoding inhibitors of the NF-κB system. In addition, tumor microenvironment-derived factors trigger aberrant activation of cytokine receptor signaling (blue rectangle) in myeloma cells. As such, genetic and microenvironmental factors activate both the NF-κB activating pathways, and interdependent regulation of these pathways promotes pathological NF-κB activity and reinforces NF-κB-driven expressions of pro-survival genes in myeloma cells.

**Concomitant activation of canonical and non-canonical NF-κB signaling in MM:** a heightened activity of the canonical NF-κB pathway has been reported in several human malignancies. In MM, both genetic mutations and microenvironmental cues trigger this pathway. Canonical signaling induces the RelA NF-κB activity, which stimulates the transcription of genes encoding pro-inflammatory cytokines and pro-survival factors. However, canonical signaling generally induces a rather transient RelA activity ex vivo owing to negative feedback regulations. Genetic lesions and to some extent, tumor microenvironmental cues additionally activate the non-canonical NF-κB pathway in myeloma cells. In fact, frequent engagement of non-canonical signaling constitutes a prominent feature of MM [65]. However, the non-canonical pathway induces a sustained RelB NF-κB activity, which mediates the expression of mostly immune differentiating and immune organogenic factors in physiological settings. Considering relatively limited physiological functions of RelB in immune maturation, a preponderance of mutations in the non-canonical pathway in MM appears somewhat puzzling. Nevertheless, mutational activations of non-canonical signaling have been described in other hematological malignancies, including chronic lymphocytic leukemia (CLL), T-cell lymphoma and cutaneous B- and T-cell lymphomas [88]. Mechanistic analyses by several groups elucidated that the canonical and the non-canonical pathways are interdependently regulated [89]. As elaborated below (Figure 3), we propose that dysfunctions of an integrated NF-κB system, and not that of the individual canonical and non-canonical pathways, cause pathological NF-κB activity in MM. In other words, an integrated NF-κB system multiplies the effect of cancer-associated non-canonical signaling and causes deregulation of several NF-κB factors. 

**RelA NF-κB activation by non-canonical signals:** Mechanistic studies involving mouse embryonic fibroblasts (MEFs) and B cells revealed that p100 retained not only RelB but also a sub-population of RelA and c-Rel subunits within the IκBsome complex [46,90,91,92]. Consistently, disruption of the IκBsome by LTβR-induced non-canonical signaling led to concomitant nuclear activation of both RelA and RelB NF-κB dimers [90]. Furthermore, NIK was shown to modulate directly the activity of the NEMO-IKK complex, which controls the canonical RelA NF-κB response [93]. Mutational activation of NIK indeed induced both canonical and non-canonical NF-κB signaling in MMCLs [94]. In comparison to targeting one or the other pathway, dual inhibition of mediators of both the pathways achieved significantly more anti-tumor activities in preclinical studies, which involved MMCLs, primary patient-derived cells, and xenograft murine models [95,96]. Therefore, it appears that NF-κB activating pathways are interlinked in MM and cooperate for promoting growth and survival of myeloma cells. 

**RelB:p50 NF-κB activation by canonical signals:** interestingly, canonical TNF signaling induced an additional, long-lasting RelB:p50 activity in *Nfkb2*^-/-^ MEFs, which lacked the expression of the non-canonical signal transducer p100 [63,97]. RelB is encoded by a NF-κB target gene [98]. Mechanistic studies clarified that RelA-dependent as well as autoregulatory mechanisms strengthened TNF-induced synthesis of RelB, which accumulated in the nucleus as an enduring NF-κB activity in the absence of the inhibitory p100 [63]. Curiously, it was demonstrated that Fbxw7 (F-box/WD40 repeat-containing protein 7), which supports the degradative K48-linked polyubiquitination of proteins, marked p100 for complete degradation in myeloma cells [99]. Likewise, mutational activation of the non-canonical pathway was associated with complete degradation of p100 in MMCLs [63]. Remarkably, NF-κB dependent RelB synthesis perpetuated a sustained RelB:p50 activity upon TNF stimulation of these myeloma cells. This TNF-induced RelB activity was implicated in the resistance of MMCLs harboring non-canonical pathway mutations to TRAIL-mediated apoptosis. Furthermore, RELB mRNA levels inversely correlated with the response of MM patients to therapeutic interventions [63]. In sum, genetic aberrations chronically activated the non-canonical pathway in myeloma cells that led to complete degradation of p100, instead of its partial processing into p52. p100-depleted malignant cells utilized the canonical TNF signal for eliciting a sustained, pro-survival NF-κB activity, which was composed of the RelB:p50 dimer and dependent on the NF-κB-driven RelB synthesis. Notably, RelA-dependent RelB synthesis was implicated in the activation of the RelB:p50 dimer in transformed B-cells [100] and invasive breast cancer cells [101]. Finally, MM-associated mutations in the non-canonical pathway seem to exacerbate the drug-resistance of malignant cells by altering the composition, and the dynamical control of the canonical NF-κB activity induced by microenvironmental cues, such as TNF. 

**Interlinked NF-κB pathways in the regulation of RelB NF-κB:** canonical signaling also modulates the RelB:p52 activity induced by non-canonical inducers. RelA upregulates the transcription of *Nfkb2*, which encodes p100 [102]. It was demonstrated that BCR-induced canonical signaling abundantly produced p100 in mature B cells, and that apt conversion of this p100 into p52 by the BAFF-stimulated non-canonical pathway generated a robust RelB:p52 response [103]. On the other hand, an absence of RelA abrogated RelB synthesis and LTβR-induced RelB activity [97]. It was also proposed that p50 and p52 were generated in mouse-derived cells through an interdependent proteasomal processing mechanism involving the respective precursor proteins p105 and p100 [97,104]. Interestingly, CD40 simultaneously activates both the NF-κB pathways and is widely expressed on myeloma cells [105,106]. Engagement of CD40 enhances adhesion of myeloma cells to extracellular matrix proteins and BMSCs, and induces the production of IL-6 and VEGF by malignant cells. In fact, anti-CD40 monoclonal antibodies have been evaluated in clinical trials involving MM patients [107]. Despite the role of CD40 in the pathogenesis of the disease, CD40-mediated regulation of the RelB NF-κB activity has not been investigated in the context of multiple myeloma. It remains unclear if CD40-activated non-canonical signaling degrades p100 completely to promote canonical RelB:p50 activation, or crosstalk between CD40-induced NF-κB pathways contributes to aberrant RelB:p52 activation myeloma cells. It is likely that other microenvironmental and genetic factors, either alone or in combination, concurrently activate both the pathways in multiple myeloma. In this context, it will be important to investigate further if ongoing canonical signaling reinforces the RelB NF-κB activity induced by the non-canonical pathway in cancerous cells. Considering that dysfunctions associated with an integrated NF-κB system appears to cause myeloma-associated inappropriate NF-κB activation, further studies ought to delineate the molecular connectivity between NF-κB pathways in diseased cells.

## 9. NF-κB Driven Gene Expressions in Myeloma Cells

Aberrant activation of the NF-κB family of transcription factors in myeloma cells causes heightened expressions of a variety of tumor-promoting cytokines, which further act in an autocrine loop on malignant cells. Clinical studies involving serum and bone marrow samples derived from MM patients revealed a substantially augmented level of TNF, IL-1, IL-6 and BAFF [108,109,110,111]. Analysis of MMCLs confirmed that myeloma cells were indeed capable of inducing the expression of genes encoding tumor-promoting cytokines. In addition, enduring NF-κB signaling stimulated cancer cell-intrinsic expressions of pro-survival factors, including cFLIP, cIAP2, Bcl-xL, Bcl2 and Gadd45β [63,65,112]. These pro-survival factors promoted myeloma growth and imparted resilience in cancerous cells from apoptosis-inducing chemotherapeutic agents. In addition, NF-κB signaling has been also implicated in the elevated expression of proto-oncogenes encoding c-Myc as well as c-Myb [113,114]. Finally, cell adhesion molecules, such as ICAM1, were produced by tumor cells in a NF-κB-dependent manner. 

It is generally thought that primarily RelA-containing heterodimers mediate the transcription of NF-κB target genes from the κB-driven promoters, while RelB heterodimers stimulate selectively the expression of immune differentiating factors. However, biophysical and biochemical analyses pointed out that RelA and RelB dimers possessed rather overlapping κB DNA binding specificities [60,61,62]. Furthermore, it was shown that a reduced p100 level in dendritic cells enabled RelB:p50 activation by canonical signaling and that the RelB:p50 heterodimer induced by the canonical pathway mediated the expression of certain RelA-target genes [64]. Subsequently, Roy et al. (2017) utilized a panel of mouse-derived knockout cells devoid of one or more NF-κB subunits to examine the gene-expression specificity of NF-κB heterodimers [63]. Their global-scale mRNA analyses established that RelB:p50 could largely substitute the RelA:p50 heterodimer for mediating TNF-induced expressions of NF-κB target genes, including those encoding pro-survival factors. Interestingly, over-expression of IKK, which selectively activates canonical signaling, or the non-canonical signal transducer NIK led to inductions of nearly overlapping set of genes in MMCLs [94]. These studies indicated that RelA and RelB heterodimers might coordinately control NF-κB driven gene-expressions in MM.

In contrast to its rather limited physiological functions, RelB was reported to play a critical role in promoting survival and drug resistance of myeloma cells. This pro-survival RelB function was attributed to RelB-dependent expressions of anti-apoptotic genes [63,65,86]. Cormier et al. (2013) demonstrated that ~40% of newly diagnosed MM patients possessed tumor cells with heightened nuclear RelB:p50 DNA binding activity. Their analysis further disclosed that RelB directly bound to the promoter of several NF-κB target pro-survival genes and activated their expressions in MMCLs [65]. In MMCLs harboring gain-of-function mutations in the non-canonical NF-κB module, TNF activated the RelB:p50 heterodimer, which similarly sustained the transcription of pro-survival genes, including *cFLIP* and *Bcl2* [63]. These studies identified RelB as a potent activator of pro-survival genes in myeloma cells. However, a recent study revealed that a HDAC4-RelB complex inhibited the transcription of pro-apoptotic gene *Bim* in MMCLs [54]. Further mechanistic investigations are warranted to unravel if RelB indeed functions as an activator and a repressor in different subsets of myeloma cells. Nevertheless, not only mutations onto the non-canonical NF-κB module are prevalent in MM, but RelB NF-κB dimers also appear to supplement pathological RelA functions in myeloma cells. Taken together, intimately interlinked NF-κB pathways and a set of NF-κB transcription factors with overlapping gene-expression specificities perpetuate pro-survival signaling in myeloma cells. 

## 10. Conclusions

Signals derived from diverse genetic and microenvironmental factors converge predominantly onto the NF-κB system in myeloma cells, highlighting the seminal role of NF-κB in the pathogenesis of MM. In myeloma cells, cell-intrinsic mutations activate mostly the non-canonical NF-κB pathway, whereas tumor microenvironment-derived cytokines trigger largely canonical signaling. Emerging evidence suggests that crosstalk between the NF-κB-activating pathways integrates mutational and microenvironmental signals to provoke anomalous NF-κB activation in MM. In particular, NIK-dependent non-canonical signaling amplifies RelA NF-κB responses and canonical signaling reinforces RelB NF-κB activity in myeloma cells. Importantly, RelB-containing heterodimers play a broader role in MM. They mediate the expression of pro-survival genes, which are traditionally thought to be RelA targets, and supplement the anti-apoptotic RelA function by protecting myeloma cells from apoptotic, chemotherapeutic drugs. We hypothesize that an integrated NF-κB system should be considered in sum, for understanding further NF-κB deregulations in MM.

The constituents of the non-canonical pathway contribute to the pro-survival and pro-proliferative program of myeloma cells *via* the integrated NF-κB system. In fact, an overarching role of non-canonical signaling in MM led to active consideration of NIK as a promising drug target [115]. Future studies should further characterize interdependent regulations of NF-κB pathways in MM, as these crosstalk connectivities may guide disease state-specific therapeutic interventions involving dual inhibition of both the NF-κB-activating pathways. Moreover, it remains unclear if the myeloma-associated RelB only activates the expression of RelA-target pro-survival genes or also mediates the expression of genes that are normally not induced by RelA. Global transcriptomic and chromatin-binding analyses involving mouse-derived knockout cells may offer valuable insights on the identity of RelA-target, RelB-target and generic NF-κB-dependent genes. These NF-κB gene signatures can be exploited for interrogating genome-scale data obtained from MM patients for assessing possible involvement of NF-κB pathways in the disease pathogenesis. Based on the outcome of such studies involving longitudinal cohorts, the NF-κB gene signatures can be further utilized as prognostic tools for predicting disease outcome, response to therapy, and the survival parameters of newly diagnosed MM patients. Furthermore, such analyses can potentially inform personalized treatment regimes for achieving efficacious and specific therapeutic interventions involving the NF-κB system. Finally, it is known that only a subset of MGUS progresses into MM. Recent high-throughput genome sequencing studies indicated that NF-κB-activating mutations are rather rare in the MGUS stage as compared to MM [116,117]. In this context, global transcriptomic analyses ought to address whether bone marrow-derived cues trigger uncontrolled NF-κB activation in MGUS and if such NF-κB deregulations precede the transition of MGUS into MM. An understanding of the mechanism that drives benign MGUS into malignant MM may enable the development of preventive measures. In conclusion, a multi-disciplinary approach, which involves mechanistic and clinical studies, will be indispensable for improving further therapeutic modalities in multiple myeloma. 

## Figures and Tables

**Table 1:** Genetic abnormalities in multiple myeloma (MM).

Genetic Abnormalities	Genes or Chromosomes Affected	Comments	References
Hyperdiploidy	chromosomes 3, 5, 7, 9, 11, 15, 19, 21	Functional role in the pathogenesis of MM remains elusive.	[12,14]
Monosomy	chromosome 13	Functional role of remains unclear.	[11]
Frequent IGH translocations	t(11;14)(q13;q32) *CCND*1 t(4;14)(p16;q32) *FGFR*3	Upregulate the expression of oncogenes encoding cyclin D1 and fibroblast growth factor receptor 3.	[15,16]
	t(4;14)(p16;q32) *MMSET*/*WHSC*1	Upregulate the expression of *MMSET*, which methylates chromatin-associated proteins and modulates their functions.	[17]
Relatively rare translocations	t(14;16)(q32;q23) *MAF* t(14;20)(q32;q11) *MAFB* t(6;14)(p21;q32) *CCND*3	These chromosomal translocations cause deregulated expressions of cell cycle regulators, including cyclin D2 and cyclin D3.	[18,19,20]
Duplication	chromosome 1 (1q)	Increased incidences in advanced MM, functional roles are unclear.	[23]
Deletions	1p, 6q, 8p, 12p, 14q, 16q, 17p, 20p	Functional role remains unclear.	[21,22]
	*BIRC*2 *BIRC*3 *TRAF*3 *CYLD*	Frequent homozygous deletions, which disrupt the function of various inhibitors of the NF-κB system.	[33,34]
	*TNFAIP*3	Heterozygous deletions, which inactivate the inhibitor of IKK, A20.	[35]
	*CDKN*2*C*	Abrogate the function of the tumor suppressor protein cyclin-dependent kinase inhibitor 2C.	[26]
Gene mutations	*NRAS* *KRAS* *BRAF*	Gain-of-function mutations in *NRAS* and *KRAS* trigger aberrant MAPK activity and are associated with the progression of MM, mutations in the *BRAF* oncogene promote myeloma growth.	[25]
	*TP*53	Abrogate the expression of p53 tumor suppressor in advanced MM.	[27]
8q24 locus rearrangements	*MYC*	Upregulate the expression of the *MYC* oncogene.	[28]
